# Cognitive Decline in Older Adults and Caregiver Distress: Stress-Burden Pathways in Dementia Care

**DOI:** 10.1093/geroni/igaf122.3317

**Published:** 2025-12-31

**Authors:** Rajib Biswas, Melinda Higgins, Fayron Epps, Kenneth Hepburn

**Affiliations:** Emory University, Atlanta, Georgia, United States; Emory University, Atlanta, Georgia, United States; UT Health San Antonio, San Antonio, Texas, United States; Emory University, Atlanta, Georgia, United States

## Abstract

This secondary analysis of the Tele-Savvy randomized trial examines pathways linking cognitive decline in older adults with dementia to caregiver psychological distress. Using baseline data from 261 caregivers (77 male, 184 female), validated measures (IQCODE, PSS, ZBI, CESD) assessed cognitive decline, stress, burden, and depression. Parallel/serial mediation analyses (PROCESS Models 4/6/85) tested stress and burden as mediators of cognitive decline’s impact on depression, with mindful self-care as a moderator. Cognitive decline correlated positively with caregiver stress (ρ = 0.24), burden (ρ = 0.31), and depression (ρ = 0.23; ps < 0.01). Stress and burden fully mediated the cognitive decline-depression pathway (total indirect effect: B = 4.38, 95% CI[2.60,6.31]), explaining 56.6% of depression variance. Female caregivers reported higher stress (d = −0.57), burden (d = −0.52), and depression (r = −0.20; ps < 0.01) than males. Mindful self-care inversely correlated with stress (ρ = −0.36), burden (ρ = −0.22), and depression (ρ = −0.29; ps < 0.01) but did not moderate mediation pathways (ps > 0.05). These findings align with the parent trial, where Tele-Savvy reduced caregiver stress and depression with moderate-to-large effects. However, burden remained unmitigated, underscoring the need for targeted strategies. Results highlight cognitive decline’s indirect effects through sequential stress-burden mechanisms, with marked gender disparities. Implications emphasize integrating gender-sensitive approaches into caregiver support programs, prioritizing stress reduction, and advancing longitudinal research to clarify causality. This work advances understanding of modifiable factors in dementia caregiving and aligns with person-centered aging frameworks, promoting equitable solutions for diverse older populations.

